# ﻿Two new species of *Micropsalliota* (Agaricales, Agaricaceae) from subtropical regions of China

**DOI:** 10.3897/mycokeys.113.140599

**Published:** 2025-02-06

**Authors:** Jun-Qing Yan, Zhi-Heng Zeng, Ya-Ping Hu, Cheng-Feng Nie, Bin-Rong Ke, Sheng-Nan Wang, Hui Zeng

**Affiliations:** 1 Institute of Edible mushroom, Fujian Academy of Agricultural Sciences, Fuzhou 350011, China Institute of Edible mushroom, Fujian Academy of Agricultural Sciences Fuzhou China; 2 Jiangxi Provincial Key Laboratory of Excavation and Utilization of Agricultural Microorganisms, Jiangxi Agricultural University, Nanchang 330045, China Jiangxi Agricultural University Nanchang China; 3 Nanjing Institute of Environmental Sciences, Ministry of Ecology and Environment Mountains, Nanjing 210042, China Nanjing Institute of Environmental Sciences, Ministry of Ecology and Environment Mountains Nanjing China

**Keywords:** Basidiomycetes, phylogeny, taxonomy, two new taxa

## Abstract

*Micropsalliota* is a relatively small genus, with 97 names recorded in previous research. In this study, two new species of *Micropsalliota*, *M.bispora* and *M.vulgaris*, have been identified based on morphological and phylogenetic evidence from subtropical regions of China. Morphologically, *M.bispora* is characterized by very small basidiomata, cymbiform basidiospores up to 9.0 μm long, white to cream pileus with fawn to dull red center, and tibiiform cheilocystidia; *M.vulgaris* is recognized by small basidiomata, medium-sized spores, white to cream pileus, covered with reddish brown to dark brown fibrils, various cheilocystidia that are up to 60 μm long, and hyphae of fibrils exhibiting pale brown vacuolar pigmentation. Their distinct taxonomic status is confirmed by the positions of the two new species in 4-locus (ITS, LSU, *rpb2*, *tef-1α*) phylogenetic trees. Detailed descriptions and morphological photographs of two new species are presented. To aid in diagnosis, a key to 35 species of *Micropsalliota* in China is provided.

## ﻿Introduction

*Micropsalliota* Höhn. was circumscribed by Höhnel in 1914 to accommodate species within *Agaricus* L. that form small and slender basidiomata (Höhnel 1914). It was once based on inappropriate characteristics and considered a dubious genus. Later, it was amended by Heinemann (1956) and Pegler and Rayner (1969), and subsequently accepted by Singer (1975). Currently, it is a widely accepted view that species of *Micropsalliota* are usually characterized by small, slender basidiomata, frequently with capitate or subcapitate cheilocystidia, pileipellis hyphae that are incrusted and turn green in ammonia solution when fresh, and, in most species, basidiospores with apically thickened endosporium and lacking germ pore (Heinemann 1977; Zhao et al. 2010).

*Micropsalliota* spp. generally grow in the tropics and subtropics, but the diversity of known species is not particularly rich. Only 97 names, including synonyms and subspecies, were listed in Index Fungorum and about 87 species are recorded worldwide (Al-Kharousi et al. 2022; Yan et al. 2022; Gao et al. 2024).

Heinemann (1983) divided the genus into four groups based on the stature of the basidiomata, the color of the pileus, and the shape of the cheilocystidia, and devised a method he called the “IG value” for measuring stature. However, Zhao et al. (2010) and Yan et al. (2022) argue that the IG value is not a reliable measure. When comparing numerous individual basidiomes from numerous populations of a single species, the IG value exhibits significant variation and often overlaps with Heinemann’s group designations. In previous molecular systematics studies, Zhao et al. (2010), Yan et al. (2022) and Gao et al. (2024) constructed its phylogeny using two-gene dataset (nrITS and nrLSU), three-gene dataset (nrITS, nrLSU, and *rpb2*), and four-gene dataset (nrITS, nrLSU, *rpb2*, and *tef-1α*), respectively. The study of Gao et al. (2024) shows that the genus is separated into 11 major clades and subclades.

In recent years, China has been a hotspot for the discovery of new species in this genus. Following the discovery of *M.pseudoglobocystis* Li Wei & R.L. Zhao as a new species, an additional 18 new species have been reported in China, bringing the total number of species to 33. (Wei et al. 2015; Li et al. 2021; Yan et al. 2022; Ji and He 2023; Gao et al. 2024). As part of the study on Chinese macrofungi species, two new species were discovered, during our investigations into subtropical regions of China. This study further elucidates the diversity of *Micropsalliota* in the area, following our publication of six new species of *Micropsalliota* from this region in 2022 (Yan et al. 2022). Detailed information on the new taxa is presented in this study.

## ﻿Materials and methods

### ﻿Morphological studies

Specimens were collected from Fujian and Zhejiang provinces of China in 2022 and were deposited in the Herbarium of Fungi, Jiangxi Agricultural University (HFJAU). Macroscopic descriptions were based on detailed field notes of fresh basidiomata and photos. Colour codes follow the Methuen Handbook of Colour (Kornerup and Wanscher 1978). Microscopic structures were observed and measured from dried specimens mounted in water and 5% KOH. Congo red was used as a stain when necessary (Horak 2005). At least 40 basidiospores, basidia, and cystidia were measured for each collection. The range of spore size is expressed in the form (a)b–c(d), where “a” and “d” represent the minimum and maximum values, respectively, 90% of the spores fall within the range ‘b–c’. The meanings of the other spore characteristics are as follows: “Q” stands for the ratio of length and width (Yan et al. 2022; Chen et al. 2024).

### ﻿DNA extraction, PCR amplification, and sequencing

DNA was extracted from dried specimens with the NuClean Plant Genomic DNA kit (CWBIO, China). Four regions (ITS, LSU, *rpb2*, *tef-1α*) were selected for the study and were amplified using the primer pairs ITS1/ITS4(White et al. 1990), LR0R/LR7 (Hopple and Vilgalys 1999), bRPB2-6F/bRPB2-7R (Matheny 2005), EF1-983F/EF1-1567R (Rehner and Buckley 2005), respectively. PCR was performed using a touchdown program for all regions: initial 95 °C for 5 min, and then 14 cycles of denaturing at 95 °C for 30 s, annealing at 65 °C for 45 s (-1 °C per cycle), extension at 72 °C for 1 min; then 30 cycles of denaturing at 95 °C for 30 s, annealing at 52 °C for 30 s, extension at 72 °C for 1 min; final extension at 72 °C for 10 min (Wang et al. 2022; Yan et al. 2022). The PCR products were sequenced by Qing Ke Biotechnology Co. Ltd (Wuhan City, China).

### ﻿Alignment and phylogenetic analyses

Sequence reads were assembled and edited using SEQUENCHER v.5.4 and were deposited in GenBank database. Based on the research by Yan et al. (2022) and Gao et al. (2024), and the similarity of these new species to the most closely related sequences identified in the BLAST results of ITS, 242 nucleotide DNA sequences in NCBI GenBank were downloaded. *Agaricuscrassisquamosus* R.L. Zhao, *A.variicystis* Linda J. Chen, K.D. Hyde & R.L. Zhao, *Hymenagaricusepipastus* (Berk. & Broome) Heinem. & Little Flower, *H.* sp. and *Leucoagaricuscentricastaneus* Y.R. Ma, Z.W. Ge & T.Z. Liu were chosen as outgroup taxa according to the results of Yan et al. (2022) and Gao et al. (2024). A total of 258 sequences including 100 ITS, 82 LSU, 46 *rpb2*, and 30 *tef1* sequences were used in subsequent analyses. Details are presented in Table 1.

**Table 1.** Details of sequences used in the phylogenetic analyses.

Taxa	Vouchers	ITS	LSU	*rpb2*	*tef-1α*	Reference
*Micropsalliotaalba*	–	EF069420	–	–	–	Gao et al. (2024)
*M.albella*	LE2016123 Holotype	MN294514	MN294516	–	–	He et al. (2020)
*M.albofelina*	LE312536 Holotype	OK257212	OK257209	–	–	Crous et al. (2021)
*M.albofelina*	HKAS70329	OR799877	OR799922	OR962218	OR962180	Gao et al. (2024)
*M.albosericea*	zrl3049	HM436644	–	–	–	Zhao et al. (2010)
*M.allantoidea*	zrl2038 Holotype	HM436648	HM436597	–	–	Zhao et al. (2010)
*M.appendiculata*	HKAS131127	OR799912	OR799956	OR962247	OR962204	Gao et al. (2024)
*M.appendiculata*	LE F-315913 Holotype	OR161109	OR161104	–	–	Ivanova et al. (2023)
*M.arginophaea*	zrl3110	HM436617	HM436577	–	–	Zhao et al. (2010)
*M.arginophaea*	HKAS60309	OR799878	OR799923	OR962219	OR962208	Gao et al. (2024)
*M.bifida*	zrl3067 Holotype	HM436640	HM436591	–	–	Zhao et al. (2010)
*M.bifida*	HFJAU2998	OM650272	OM650252	OM669858	–	Yan et al. (2022)
*M.bispora*	HFJAU3833	PQ345346	PQ345351	PQ358515	PQ358519	this study
*M.bispora*	HFJAU4253 Holotype	PQ345347	PQ345352	PQ358516	PQ358520	this study
*M.brunneosquamata*	LD201236 Holotype	KP316210	–	–	–	Chen et al. (2016)
*M.cortinata*	zrl2129	HM436630	HM436593	–	–	Zhao et al. (2010)
*M.cortinata*	HKAS92221	OR799879	OR799924	OR962220	OR962183	Gao et al. (2024)
*M.delicatula*	HKAS54332	OR799880	OR799925	OR962221	OR962209	Gao et al. (2024)
*M.delicatula*	ZRL2015234 Holotype	MT671229	–	–	–	Li et al. (2021)
*M.dentatomarginata*	GX20170202 Holotype	MT671228	MT671242	–	–	Li et al. (2021)
*M.digitatocystis*	HKAS123832	OR799883	OR799928	OR962224	OR962185	Gao et al. (2024)
*M.digitatocystis*	ZRL20180564 Holotype	MT671239	MT671250	–	–	Li et al. (2021)
*M.ferruginea*	HKAS 131130	OR799885	OR799930	OR962226	OR962182	Gao et al. (2024)
*M.ferruginea*	HKAS 70562 Holotype	OR799884	OR799929	OR962225	OR962181	Gao et al. (2024)
*M.fimbriata*	HKAS 60241 Holotype	OR799886	OR799931	OR962227	OR962198	Gao et al. (2024)
*M.fimbriata*	HKAS 60261	OR799887	OR799932	–	OR962199	Gao et al. (2024)
*M.furfuracea*	zrl3006 Holotype	HM436621	HM436603	–	–	Zhao et al. (2010)
*M.furfuracea*	HKAS60229	OR799889	OR799934	OR962229	OR962201	Gao et al. (2024)
*M.geesterani*	LAPAG520	KM923965	KM923966	–	–	Parra et al. (2016)
*M.geesterani*	E.C. Vellinga 2263(L)	AF482857	AF482888	–	–	Parra et al. (2016)
*M.gigaspora*	HKAS131118	OR799890	OR799935	OR962230	–	Gao et al. (2024)
*M.gigaspora*	HKAS131119 Holotype	OR799891	OR799936	OR962231	–	Gao et al. (2024)
*M.globocystis* 1	HKAS131120	OR799892	OR799937	–	OR962186	Gao et al. (2024)
*M.globocystis* 1	HFJAU1518	OM650277	OM650255	OM669852	–	Yan et al. (2022)
*M.globocystis* 2	HKAS131133	OR799895	OR799940	OR962234	OR962197	Gao et al. (2024)
*M.globocystis* 2	HKAS92202	OR799894	OR799939	OR962233	OR962196	Gao et al. (2024)
*M.globocystis* 3	VDW1278	MT304640	–	–	–	Yan et al. (2022)
*M.globocystis* 4	zrl2049	HM436635	–	–	–	Zhao et al. (2010)
*M.globocystis* 4	HFJAU2709	OM650278	OM650262	OM669856	–	Yan et al. (2022)
*M.gracilis*	zrl2041	HM436647	HM436583	–	–	Zhao et al. (2010)
*M.gracilis*	HNL503432	MW192914	–	–	–	Yan et al. (2022)
*M.inflata*	LE F-315912 Holotype	OR161110	OR161106	–	–	Ivanova et al. (2023)
*M.jiangxiensis*	THJ20018	ON117420	ON117438	–	–	Ji and He (2023)
*M.jiangxiensis*	THJ20019A	ON117421	ON117439	–	–	Ji and He (2023)
M.lateritiavar.vinaceipes	zrl2073 Holotype	HM436631	–	–	–	Zhao et al. (2010)
M.lateritiavar.vinaceipes	HKAS131124	OR799896	OR799941	OR962235	OR962202	Gao et al. (2024)
*M.longicystis*	HKAS131121 Holotype	OR799897	OR799942	OR962257	–	Gao et al. (2024)
*M.longicystis*	HKAS131126	OR799898	OR799943	OR962258	–	Gao et al. (2024)
*M.megarubescens*	zrl2086 Holotype	HM436620	–	–	–	Zhao et al. (2010)
*M.megarubescens*	HKAS60253	OR799900	OR799945	OR962237	OR962189	Gao et al. (2024)
*M.megaspora*	zrl3068 Holotype	HM436624	–	–	–	Zhao et al. (2010)
*M.megaspora*	HFJAU1255	OM650282	OM650258	OM669876	–	Yan et al. (2022)
*M.minor*	HFJAU2796	OM650294	OM650266	OM669865	–	Yan et al. (2022)
*M.minor*	HFJAU2812 Holotype	OM650293	–	OM669864	–	Yan et al. (2022)
*M.nana*	HKAS114619	OR799901	OR799946	OR962238	OR962216	Gao et al. (2024)
*M.nana*	HKAS115226 Holotype	OR799902	OR799947	–	OR962217	Gao et al. (2024)
*M.ovalispora*	HFJAU2010 Holotype	OM650295	OM650269	OM669866	–	Yan et al. (2022)
*M.ovalispora*	HFJAU3179	OM650296	–	OM669867	–	Yan et al. (2022)
*M.pileocystidiata*	AMH9975 Holotype	MG917970	–	–	–	Patil et al. (2022)
*M.pileocystidiata*	MMH1114	MZ598496	–	–	–	Patil et al. (2022)
*M.pleurocystidiata*	zrl2023	HM436636	–	–	–	Zhao et al. (2010)
*M.pseudoarginea*	HKAS131125	OR799903	OR799948	OR962239	OR962212	Gao et al. (2024)
*M.pseudoarginea*	HFJAU2122	OM650284	OM650260	OM669861	–	Yan et al. (2022)
*M.pseudodelicatula*	HKAS131129	OR799905	OR799950	OR962241	OR962213	Gao et al. (2024)
*M.pseudodelicatula*	HFJAU2228 Holotype	OM650288	OM650264	OM669863	–	Yan et al. (2022)
*M.pseudoglobocystis*	HKAS87127	OR799908	OR799953	OR962244	OR962190	Gao et al. (2024)
*M.pseudoglobocystis*	ZRL2013321 Holotype	KM889913	–	–	–	Wei et al. (2015)
*M.purpureobrunneola*	LE2016124 Holotype	MN294513	MN294517	–	–	He et al. (2020)
*M.pusillissima*	zrl3047 Holotype	HM436645	HM436594	–	–	Zhao et al. (2010)
*M.repanda*	LAPAF8	KP739805	KP739804	–	–	Parra et al. (2016)
*M.roseipes*	HFJAU2494	OM650297	OM650270	OM669870	–	Yan et al. (2022)
*M.rubrobrunnescens*	zrl2120 Holotype	HM436628	HM436588	–	–	Zhao et al. (2010)
*M.rubrobrunnescens*	HKAS96929	OR799914	OR799958	OR962249	OR962206	Gao et al. (2024)
M.rubrobrunnescensvar.tibiicystis	zrl2121 Holotype	HM436629	HM436589	–	–	Zhao et al. (2010)
*M.rufosquarrosa*	HFJAU1208	OM650291	OM650267	OM669868	–	Yan et al. (2022)
*M.rufosquarrosa*	HFJAU1236 Holotype	OM650292	OM650268	OM669869	–	Yan et al. (2022)
*M.squarrosa*	HKA128713	OR799916	OR799960	OR962251	–	Gao et al. (2024)
*M.squarrosa*	HKAS128633 Holotype	OR799915	OR799959	OR962250	–	Gao et al. (2024)
*M.subalba*	zrl2080	HM436646	HM436596	–	–	Zhao et al. (2010)
*M.subalba*	HKAS105828	OR799917	OR799961	–	OR962211	Gao et al. (2024)
*M.subarginea*	zrl2052	HM436612	HM436573	–	–	Zhao et al. (2010)
*M.subarginea*	zrl2092	HM436611	HM436574	–	–	Zhao et al. (2010)
*M.suricatoides*	LE F-348071	OR161111	OR161105	–	–	Ivanova et al. (2023)
*M.suricatoides*	LE F-348072 Holotype	OR161112	–	–	–	Ivanova et al. (2023)
*M.tenuipes*	HFJAU1536 Holotype	OM650289	–	–	–	Yan et al. (2022)
*M.tenuipes*	HFJAU3180	OM650290	OM650265	–	–	Yan et al. (2022)
*M.umbonata*	HKAS131131 Holotype	OR799920	OR799964	OR962254	OR962194	Gao et al. (2024)
*M.umbonata*	HKAS131132	OR799921	OR799965	OR962255	OR962195	Gao et al. (2024)
*M.ventricocystidiata*	SQUH-ATR004	OM397373	OM630413	–	–	Al-Kharousi et al. (2022)
*M.ventricocystidiata*	SQUH-GOB002 Holotype	OM397374	OM630414	–	–	Al-Kharousi et al. (2022)
*M.vulgaris*	HFJAU3350 Holotype	PQ345344	PQ345349	PQ358513	PQ358517	this study
*M.vulgaris*	HFJAU5707	PQ345345	PQ345350	PQ358514	PQ358518	this study
*M.wuyishanensis*	HFJAU3048 Holotype	OM650298	–	OM669878	–	Yan et al. (2022)
*M.xanthorubescens*	zrl3083	HM436638	HM436598	–	–	Zhao et al. (2010)
*M.xanthorubescens*	NW1356	MW504965	–	–	–	Yan et al. (2022)
**Outgroup**						
*Agaricuscrassisquamosus*	ZRL2012607 Holotype	KT951376	KT951510	–	–	Zhao et al. (2016)
*A.variicystis*	LD201234 Holotype	KT951339	KT951517	–	–	Zhao et al. (2016)
*Hymenagaricusepipastus*	ZRL3045	HM436649	HM436609	–	–	Zhao et al. (2010)
*Hymenagaricus* sp.	ZRL3103	KM982450	KM982452	–	–	Li et al. (2021)
*Leucoagaricuscentricastaneus*	SYAU FUNGI 076 Holotype	OM976855	OM976871	OR962256	OR962207	Gao et al. (2024)

**Figure 1.**
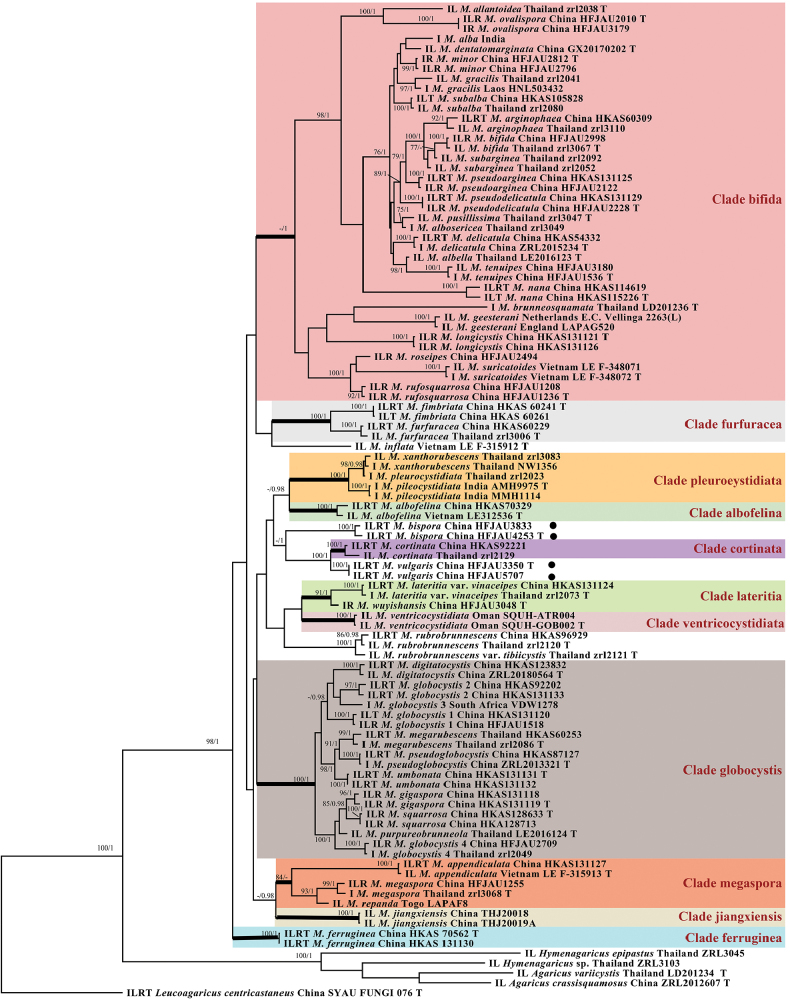
Phylogram of *Micropsalliota* generated by Bayesian inference (BI) analysis based on sequences of ITS(I)+LSU(L)+*rpb2* (R)+*tef-1α* (T). It was rooted with *Agaricus* spp. *Hymenagaricus* spp. and. *Leucoagaricuscentricastaneus*. Posterior probabilities (BI-PP) and ML bootstrap values (ML-BP) ≥ 0.95 and ≥75%, respectively, are shown as BP/PP. Black bullet indicates newly described taxa. T indicates Holotype.

Sequence datasets, containing intron regions, were separately aligned on the MAFFT v.7 (Katoh and Standley 2013). Bayesian Inference (BI) and Maximum Likelihood (ML) phylogenetic analyses of the aligned concatenated dataset were carried out in MRBAYES v.3.2.7a and RAXML 8.2.12, respectively (Ronquist and Huelsenbeck 2003; Stamatakis 2006). The best-fit models of BI were determined by PARTITIONFINDER, based on the Corrected Akaike information criterion (AICc) (Lanfear et al. 2017). The GTRGAMMA model as the best-fit likelihood model for ML analysis with 1,000 replicates and allowing partitions to have different seeds (Silvestro and Michalak 2012). For the Bayesian analysis, four Monte Carlo Markov chains were run for 10 million generations, sampling every 1000^th^ generation, with the first 25% of trees discarded as burn-in. Branches with Bayesian posterior probability (BI-PP) ≥ 0.95 and ML bootstrap support (ML-BP) ≥ 75% are considered statistically supported and are shown in the tree (Fig. 1). All alignments for phylogenetic analyses and the resulting trees were deposited in TreeBASE (ID: 31925, http://purl.org/phylo/treebase/phylows/study/TB2:S31925?x-access-code=2021c0bf9e5d391e2fa622a887e944b3&format=html).

## ﻿Results

A total of 2708 characters from 100 taxa were used in phylogenetic analyses (ITS 774 bp; LSU 752 bp; *rpb2* 608 bp; *tef-1α* 574 bp), of which 403, 133, 248, 299 sites were variable and 304, 106, 223, 188 sites were parsimony informative for ITS, LSU, *rpb2*, *tef-1α*, respectively. The best models are calculated separately, and the results are as follows: the best models for Bayesian analysis were GTR+I+G for the ITS and LSU, SYM+I+G for the *rpb2* and *tef-1α*. The loglikelihood of the ML consensus tree was -21457.61, and the average standard deviation of split frequencies less than 0.01 after 1.88 million generations in the Bayesian analysis.

As shown in the Bayes tree, the 11 major clades and subclades proposed by Gao et al. (2024) were well reproduced in this study, except for Clade lateritia. Two new species were formed distinct and stable branches, respectively, and group together with *M.cortinata* (Heinem.) Heinem., with high statistical support in the Bayesian inference (BPP = 1). Details are presented in Fig. 1.

### ﻿Taxonomy

#### ﻿ Micropsalliota bispora

Taxon classificationFungiAgaricalesAgaricaceae

J.Q. Yan, S.N. Wang, & H. Zeng sp. nov.

87367F77-7D61-510A-859F-DB08B559AC4D

 855802

Fig. 2

##### Etymology.

Name refers to the majority of basidia are 2-spored.

##### Diagnosis.

*Micropsalliotabispora* is mainly characterized by very small basidiomata; white to cream pileus, with the center being fawn to dull red; cymbiform basidiospores in profile view, 8.0–9.0(9.5) × 4.0–5.0(5.3) μm; and tibiiform cheilocystidia. It differs from *M.albofelina* by having bigger spores, which are up to 9.0 μm in length.

**Figure 2.**
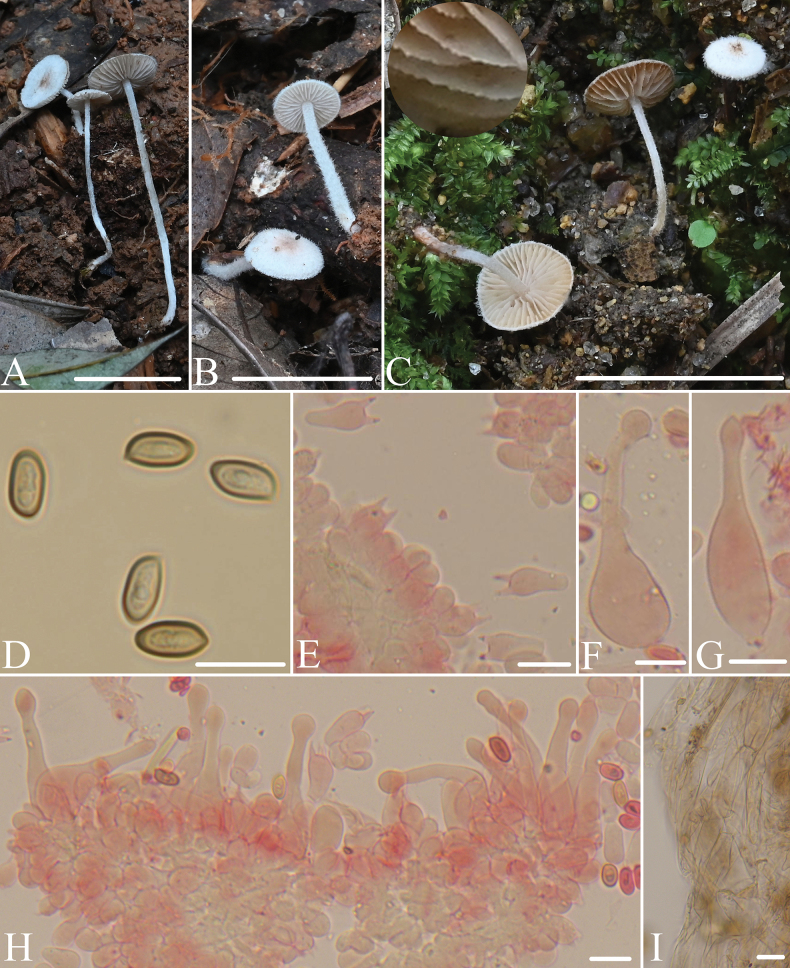
Morphological structures of *Micropsalliotabispora*. **A–C** Basidiomata **D** spores **E** basidia **F–H** cheilocystidia **I** hyphae of fibrils. All microstructures were observed in 5%KOH, structures of **E–H** were stained by 1%Congo red. Scale bars: 10 mm (**A–C**);10 μm (**D–I**).

##### Holotype.

China • Fujian Province, Wuyishan National Park, 11 July 2022, Jun-Qing Yan, Cheng-Feng Nie, HFJAU4253.

##### Description.

Pileus 3.0–5.0 mm in diameter, white to cream, fawn (7E4) to dull red (8C3) at center, plano-convex to plane, surface dry, covered with white well-developed fibrils at early age, gradually disappears with aging. Context less than 0.8 mm thick. Lamellae 0.5–1.0 mm broad, free to short adnexed, distant, white, becoming brownish orange to light brown (7C4–7D4) as mature, edge white, smooth to serrate, with two series of lamellulae. Stipe 10–25 mm long, 0.5–1.0 mm thick, cylindrical, slender, white, surface with white fibrils, gradually disappears with aging. Annulus unobserved.

Basidiospores 8.0–9.0(9.5) × 4.0–5.0(5.3) μm, Q = (1.6)1.7–2.0, cymbiform in profile view, ellipsoid to elongated-ellipsoid in face view, light brown, wall 0.5 μm thick, apically thickened endosporium, without germ pore, inamyloid. Basidia 11–15 × 6.5–8.0 μm, clavate, hyaline, 2-spored, rarely 4-spored. Pleurocystidia absent. Cheilocystidia 35–55 × 6.8–15(17) μm, tibiiform, apex capitate, rarely subacute, 4.0–6.0(7.0) μm in diameter. Fibrils at the center of pileus composed of hyphae, 8.0–15 μm broad, with pale brown membranous pigment, constricted at the septa on some hyphae.

##### Habit and habitat.

Scattered on soil in broad-leaved forest or mixed coniferous and broad-leaved forests.

##### Additional specimens examined.

China • Fujian Province, Wuyishan National Park, 25 June 2022, Jun-Qing Yan, Bin-Rong Ke, HFJAU3833.

##### Note.

Macroscopically, *M.bispora* is very similar to *M.albofelina* D.D. Ivanova & O.V. Morozova, with both species having very small basidiomata and well-developed scales or fibrils at an early age. However, the latter has spores that are shorter than 7.5 μm and a white center on the pileus (Crous et al. 2021). *Micropsalliotalongicystis* T. Gao & Z.W. Ge and *M.pseudoarginea* Heinem. are also similar to *M.bispora* macroscopically, but *M.longicystis* has pleurocystidia and its spores shorter than 6.0 μm (Gao et al. 2024), and *M.pseudoarginea* has broadly clavate or ventricose-clavate cheilocystidia and its spores shorter than 5.0 μm (Zhao et al. 2010). Among the known species of *Micropsalliota* with a pileus generally less than 10 mm, there are no species like *M.bispora* with spores longer than 8.0 μm. Based solely on this characteristic, *M.bispora* can be distinctly differentiated from known species of *Micropsalliota*.

*M.geesterani* (Bas & Heinem.) R.L. Zhao & L.A. Parra, *M.gigaspora* T. Gao & Z.W. Ge, and *M.ventricocystidiata* Al-Sadi & S. Hussain are similar to *M.bispora*, with spores up to 9.0 μm, but their pilei are larger than 20 mm. Additionally, *M.geesterani* has a purple pileus, and fusiform, cylindrical, or narrowly clavate cheilocystidia (Parra et al. 2016), *M.gigaspora* has clavate cheilocystidia (Gao et al. 2024), and *M.ventricocystidiata* has ventricose cheilocystidia (Al-Kharousi et al. 2022).

#### ﻿ Micropsalliota vulgaris

Taxon classificationFungiAgaricalesAgaricaceae

J.Q. Yan, S.N. Wang, & H. Zeng sp. nov.

66229DB0-8F78-54E6-8D03-4263A6E134B5

 855803

Fig. 3

##### Etymology.

Name refers to the fact that many known species in this genus share similar macroscopic characteristics with the new species.

##### Diagnosis.

*Micropsalliotavulgaris* is mainly characterized by small basidiomata; white to cream pileus, covered with reddish brown to dark brown fibrillose; elongated-ellipsoid to elongated basidiospores in profile view, (6.3)6.7–8.0 × 3.7–4.4(4.7) μm; various cheilocystidia; hyphae of fibrils have pale brown vacuolar pigment. It differs from *M.squarrosa* by having various cheilocystidia.

**Figure 3.**
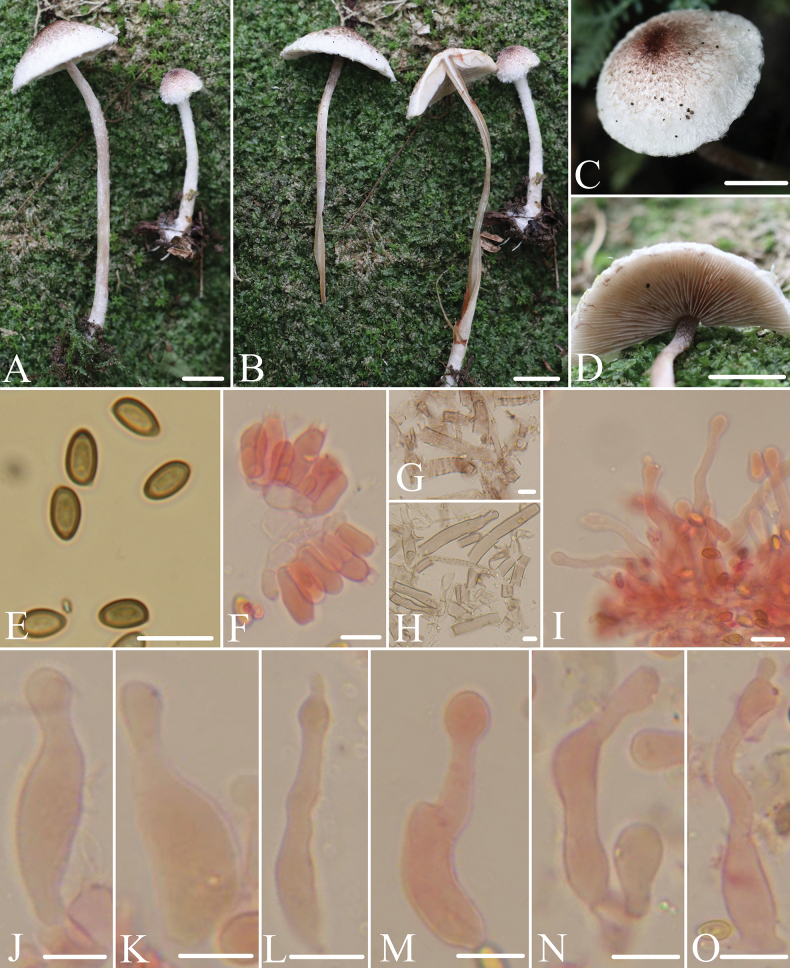
Morphological structures of *Micropsalliotavulgaris*. **A–D** Basidiomata **E** spores **F** basidia **G, H** hyphae of fibrils **I–O** cheilocystidia. All microstructures were observed in 5%KOH, except for **G**, which were observed in water. Structures of **F** and **I–O** were stained by 1% Congo red. Scale bars: 10 mm (**A–D**);10 μm (**E–O**).

##### Holotype.

China • Zhejiang Province, Wencheng County, 24 May 2022, Ya-Ping Hu, Jun-Qing Yan, HFJAU3350.

##### Description.

Pileus 10–35 mm, convex, white to cream, covered with reddish brown to dark brown (9E6–9F6) fibrillose, dense at the center, sparser towards the edge. Context less than 2.0 mm thick, turning brownish red (9C7–9C8) in some areas when bruised or cut. Lamellae 3.0–4.0 mm, crowded, free, with 2–3 series of lamellulae, dull white, edge dull white, slightly serrate. Stipe 50–80 mm long, 3.0–4.0 mm thick, cylindrical, slender, white, surface with white fibrils, gradually disappears with aging. Context of stipe turning brownish red (9C7–9C8) in some areas when bruised or cut. Annulus unobserved.

Basidiospores (6.3)6.7–8.0 × 3.7–4.4(4.7) μm, Q = (1.5)1.6–1.9(2.0), elongated-ellipsoid to elongated, slightly flattened on one side in profile view, ellipsoid to elongated-ellipsoid in face view, light brown, wall 0.5 μm thick, apically thickened endosporium, without germ pore, inamyloid. Basidia 15–19 × 6.0–7.5 μm, clavate, hyaline, 4-spored. Pleurocystidia absent. Cheilocystidia 35–62 × 4.2–11 μm, various, tibiiform, capitate, subhypoid, rarely fork, apex capitate, rarely mucronate, 4.0–8.0 μm in diameter. Fibrils composed of hyphae 8.0–15 μm broad, with pale brown vacuolar pigment.

##### Habit and habitat.

Scattered on soil in broad-leaved forest.

##### Additional specimens examined.

China • Zhejiang Province, Wencheng County, 25 May 2022, Zhi-Heng Zeng, Shen-Nan Wang, HFJAU5707.

##### Note.

Among the known species of the *Micropsalliota*, few have a similar combination of characteristics as *M.vulgaris*, that is, a white pileus covered with brown fibrils, lacks pleurocystidia, and has spores up to 8.0 μm. However, they can be clearly distinguished from *M.vulgaris*: *M.endophaea* Heinem., *M.inflata* D.D. Ivanova & O.V. Morozova,and *M.megaspora* R.L. Zhao, Desjardin, Soytong & K.D. Hyde have pilei that are generally less than 10 mm in diameter, and their cheilocystidia are shorter than 40 μm (Heinemann 1988; Zhao et al. 2010; Ivanova et al. 2023); *M.fimbriata* T. Gao & Z.W. Ge, and *M.gigaspora* have clavate cheilocystidia and incrusted hyphae of pileus squamules (Gao et al. 2024); *M.geesterani* has a purple pileus that can reach up to 200 mm in diameter (Parra et al. 2016); *M.squarrosa* T. Gao & Z.W. Ge has clavate to clavate-capitate cheilocystidia (Gao et al. 2024); *M.ventricocystidiata* has ventricose cheilocystidia (Al-Kharousi et al. 2022).

## ﻿Discussion

In the phylogenetic tree (Fig. 1), *M.cortinata* groups together with two new species, and is very close to *M.vulgaris*. In previous studies, the branch where *M.cortinata* is located was designated as clade cortinata, which is distinguished by cortinate partial veil that leaves remnants only on the pileus margin (Zhao et al. 2010; Gao et al. 2024). The two new species, however, exhibit developed fibrils on the pileus margin, a feature that distinguishes them from clade cortinata. Additionally, the branch where these three species are located was difficult to morphologically characterize. The basidiospores of *M.cortinata* are shorter than 6.5 μm, and the cheilocystidia are clavate to ventricose, the hyphae of pileipellis have membranous pigments, which allows it to be distinctly differentiated from *M.vulgaris* (Heinemann 1980; Zhao et al. 2010). *M.bispora*, with its very small basidiomata, mostly 2-spored basidia, and tibiiform cheilocystidia, can be distinctly differentiated from *M.vulgaris*. While all three species lack pleurocystidia, this characteristic alone is not sufficient to unite them.

In previous study (Gao et al. 2024), Clade lateritia includes six species: *M.inflata*, M.lateritiavar.vinaceipes R.L. Zhao, Desjardin, Soytong & K.D. Hyde, *M.rubrobrunnescens* R.L. Zhao, Desjardin, Soytong & K.D. Hyde, M.rubrobrunnescensvar.tibiicystis R.L. Zhao, Desjardin, Soytong & K.D. Hyde, *M.suthepensis* R.L. Zhao, Desjardin, Soytong & K.D. Hyde, and *M.wuyishanensis* J.Q. Yan. However, stable support was only obtained in the Bayesian inference, and there were no clear shared characteristics. Due to *M.suthepensis* having only LSU sequences and significant morphological differences with two new species, it was not included in this phylogenetic analysis. In this study, the remaining species did not form stable clades, except for M.lateritiavar.vinaceipes and *M.wuyishanensis*, which grouped together to form a stable clade with good morphological consistency, such as ellipsoid to amygdaliform spores measuring 5–6 μm in length, red-brown squamulose pileipellis, and hyphae of fibrils exhibiting vacuolar pigments. During the research process of this genus, many issues regarding the stability of the branches were not well resolved. However, as more species are discovered, we believe that more well-supported clades will emerge in the phylogenetic tree.

### ﻿Key to *Micropsalliota* species distributed in China

**Table:** 

1	Pileus white to dirty white	**2**
–	Pileus distinctly coloured or covered with numerous coloured fibrils	**16**
2	Basidiospores mainly shorter than 5.5 μm	**3**
–	Basidiospores mainly longer than 5.5 μm	**7**
3	Cheilocystidia bifid with two toe-like subcapitate lobes	***M.bifida***
–	Not as above	**4**
4	Basidiospores ovoid in face view, amygdaliform in profile view	***M.ovalispora***
–	Not as above	**5**
5	Cheilocystidia non-capitate	***M.pseudoarginea***
–	Cheilocystidia tibiiform or lageniform, capitate at apex	**6**
6	Pileus < 5 mm in diameter, hyphae of pileus squamules hyaline	***M.pseudodelicatula***
–	Pileus >5 mm in diameter, hyphae of pileus squamules has membranous pigments	***M.nana***
7	Pileus mainly <10 mm in diameter	**8**
–	Pileus mainly >10 mm in diameter	**12**
8	Cheilocystidia two types, tibiiform or forked with capitate or subacute apex	***M.minor***
–	Not as above	**9**
9	Cheilocystidia utriform	***M.tenuipes***
–	Cheilocystidia capitate at apex	**10**
10	Spores 8.0–9.0 μm	***M.bispora***
–Spores shorter than 8.0 μm	**11**
11	Stipe less than 20 mm long, and annulus superior membranous	***M.delicatula***
–	Stipe up to 30 mm long, and annulus absent	***M.albofelina***
12	Pileus 20–80 mm in diameter	***M.megarubescens***
–	Pileus mainly <20 mm in diameter	**13**
13	Pleurocystidia present	***M.longicystis***
–	Pleurocystidia absent	**14**
14	Pileipellis hyphae with incrusted pigments	***M.dentatomarginata***
–	Pileipellis hyphae with vacuolar pigments	**15**
15	Pileus not stains	***M.subalba***
–	Pileus stains reddish brown when bruised or cut	***M.rubrobrunnescens***
16	Pleurocystidia present	**17**
–	Pleurocystidia absent	**18**
17	Cheilocystidia cylindrical to subclavate	***M.digitatocystis***
–	Cheilocystidia pyriform to subglobose	***M.appendiculata***
18	Pileus mainly <10 mm in diameter	**19**
–	Pileus larger than 10 mm in diameter	**22**
19	Pileus brown to dark brown	***M.megaspora***
–	Pileus pink, red to violet-red	**20**
20	Cheilocystidia hyphoid, often forked, up to 60 μm long	***M.wuyishanensis***
–	Not as above	**21**
21	Stipe dirty white with pink tone, cheilocystidia without deposit	**M.cf.roseipes**
–	Stipe white, cheilocystidia covered by light brown deposit	***M.rufosquarrosa***
22	Pileus >20 mm in diameter	**23**
–	Pileus <20 mm in diameter	**31**
23	Basidiospores less than 6.0 μm long	***M.pseudoglobocystis***
–	Basidiospores up to 7.0 μm long or longer	**24**
24	Stipe less than 30 mm long, cheilocystidia cylindrical to subclavate, 12–33 μm long	***M.jiangxiensis***
–	Stipe up to 50 mm long or longer, cheilocystidia not as above	**25**
25	Basidiospores mainly wider than 4.0 μm	**26**
–	Basidiospores mainly narrower than 4.0 μm	**28**
26	Spores up to 9.0 μm, cheilocystidia broadly clavate to clavate	***M.gigaspora***
–	Spores mainly less than 8.0 μm, cheilocystidia not as above	**27**
27	Cheilocystidia clavate to clavate-capitate	***M.squarrosa***
–	Cheilocystidia various, tibiiform, capitate, subhypoid, rarely fork	***M.vulgaris***
28	Flesh stains red when bruised or cut	***M.furfuracea***
–	Flesh stains yellow, reddish-brown or blue when bruised or cut	**29**
29	Flesh stains blue	***M.ferruginea***
–	Flesh stains yellow or reddish-brown	**30**
30	Pileus has conspicuously obtuse umbo; squamules light brown, yellowish brown, or brown	***M.umbonata***
–	Pileus has slightly or not umbonate, squamules purple to purplish brown, greyish brown or reddish-brown	***M.globocystis***
31	Veil cortinate	***M.cortinata***
–	Veil membranous	**32**
32	Basidiomata brown	**33**
–	Basidiomata violet-red	**34**
33	Spores shorter than 5.5 μm	***M.arginophaea***
–	Spores up to 7.5 μm	***M.fimbriata***
34	Cheilocystidia broadly clavate or seldom subcapitate	**M.lateritiavar.vinaceipes**
–	Cheilocystidia versiform, ventricose to irregularly tibiiform, capitate or subcapitate with long narrow neck	***M.gracilis***

## Supplementary Material

XML Treatment for Micropsalliota bispora

XML Treatment for Micropsalliota vulgaris

## ﻿Additional information

### Author ORCIDs

Jun-Qing Yan https://orcid.org/0000-0003-1128-5171

Sheng-Nan Wang https://orcid.org/0000-0003-0648-271X

Hui Zeng https://orcid.org/0000-0003-2025-844X

### Data availability

All of the data that support the findings of this study are available in the main text.
